# C-reactive protein flare: a promising prognostic predictor for patients with hepatocellular carcinoma treated with TACE combined with lenvatinib and immune checkpoint inhibitors

**DOI:** 10.3389/fimmu.2025.1657733

**Published:** 2025-11-11

**Authors:** Xinge Li, Xu Chang, Huiyong Wu, Jianjun Han, ChunXue Wu

**Affiliations:** 1 Department of Oncology, Central Hospital Affiliated to Shandong First Medical University, Shandong First Medical University, Shandong Academy of Medical Sciences, Jinan, Shandong, China; 2 Department of Interventional Therapy II, Shandong Cancer Hospital and Institute, Shandong First Medical University and Shandong Academy of Medical Sciences, Jinan, Shandong, China; 3 Department of Interventional Therapy III, Shandong Cancer Hospital and Institute, Shandong First Medical University and Shandong Academy of Medical Sciences, , Jinan, Shandong, China; 4 Shandong Cancer Hospital and Institute, Shandong First Medical University and Shandong Academy of Medical Sciences, Jinan, Shandong, China

**Keywords:** hepatocellular carcinoma, C-reactive protein kinetics, transarterial chemoembolization, lenvatinib, immune checkpoint inhibitors

## Abstract

**Purpose:**

To assess the prognostic value of C-reactive protein (CRP) kinetics in patients with hepatocellular carcinoma (HCC) treated with transarterial chemoembolisation (TACE) combined with lenvatinib (LEN) and immune checkpoint inhibitors (ICIs).

**Methods:**

This study retrospectively analysed CRP kinetics for 143 HCC patients treated with TACE–LEN–ICIs from December 2020 to February 2024. Initially, patients were classified into three groups based on early CRP kinetics: (i) CRP flare-responders, whose levels increased to more than double the baseline value within 1 month of the initial TACE–LEN–ICIs regime followed by a decrease to below baseline within 3 months; (ii) CRP responders, where levels decreased by at least 30% from baseline within 3 months without an initial flare; and (iii) CRP non-responders (comprising all remaining patients). Overall survival (OS), progression-free survival (PFS), objective response rate (ORR), and disease control rate (DCR) were calculated as oncological outcomes. A correlation between these outcomes and CRP kinetics was examined in this study.

**Results:**

The CRP flare-responder, CRP responder, and CRP non-responder groups included 19 (13.3%), 60 (42.0%), and 64 (44.7%) patients, respectively, exhibiting ORRs of 78.9%, 78.4% and 48.4% (*p* = 0.001) and DCRs of 94.8%, 96.7% and 78.1% (*p* = 0.004). Median PFS for the three respective groups was 40.3, 11.4, and 5.5 months (*p* < 0.001), while median OS values were not reached, 18.9 and 9.9 months (*p* < 0.001). Moreover, CRP responder and CRP flare-responder status were independent risk factors for OS (*p* < 0.001) and PFS (*p* < 0.001).

**Conclusion:**

CRP kinetics may have predictive value for prognosis in HCC patients undergoing TACE–LEN–ICIs.

## Graphical Abstract

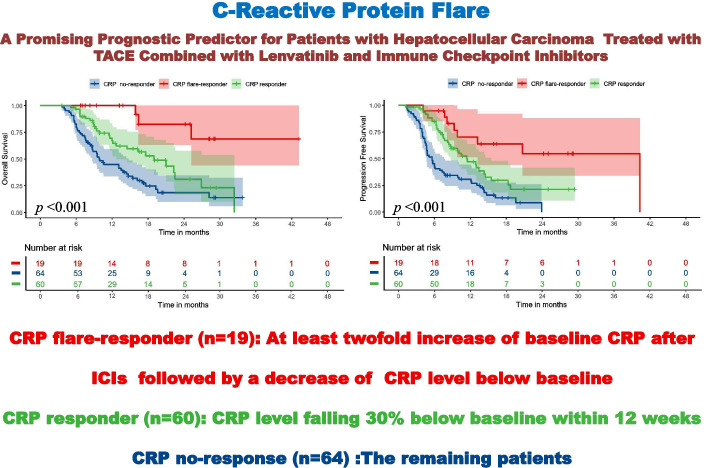

## 1 Introduction

Hepatocellular carcinoma (HCC) is one of the most prevalent malignancies and one of the leading causes of cancer-related deaths worldwide (1). In accordance with the World Health Organization (WHO), primary liver cancer (75–85% of all HCC cases) has the sixth-highest incidence globally and accounts for the third largest number of cancer deaths (1, 2). Due to its insidious onset, high invasiveness, rapid progression, and difficulties in early diagnosis, most HCC cases in China are diagnosed at locally advanced stages or with distant metastases, ruling out opportunities for surgical resection (3).

For patients with unresectable HCC (uHCC), transarterial chemoembolisation (TACE) is recommended as an effective palliative therapy that has shown encouraging survival outcomes (4). In particular, the LAUNCH study investigated TACE combined with systemic lenvatinib (LEN) treatment in uHCC and found improved efficacy and patient benefit compared with LEN monotherapy (5). Additionally, immunotherapy with immune checkpoint inhibitors (ICIs) has also been demonstrated to be effective in the treatment of HCC. In accordance with the phase III IMbrave150 study, the US Food and Drug Administration (FDA) approved atezolizumab (an anti-PD-L1 antibody) combined with bevacizumab for patients with systemic treatment-naïve, unresectable, or metastatic HCC (6).

There are a few other combination immunotherapies that have recently been developed as first-line options for HCC, including LEN with pembrolizumab (KEYNOTE-524) (7), rivoceranib with camrelizumab (CARES-310) (8), and sintilimab plus a bevacizumab biosimilar (ORIENT-32) (9). A notable example is the CHANCE001 study which found that TACE combined with PD-(L)1 inhibitors and tyrosine kinase inhibitors(TKI) showed considerable benefit in progression free survival (PFS), overall survival (OS), and objective response rate (ORR) compared with TACE monotherapy, as well as having an acceptable safety profile (10, 11). The combination of TACE with anti-PD-(L)1 antibodies and molecular targeted therapies (MTT) has been widely adopted especially in China, and its effectiveness has been demonstrated by a number of subsequent studies (10, 12–14). However, while it is crucial to identify patient populations that will benefit before undergoing this combination therapy, how to accomplish this remains uncertain. Several biomarkers have been explored to predict the immunotherapy efficacy. But to date, there is no validated biomarker that can be used in clinical decision-making. C-reactive protein (CRP) is an acute-phase reactant that indicates systemic inflammation (15). Recently, Fukuda et al. applied a novel CRP kinetics approach to nivolumab immunotherapy in metastatic renal cell carcinoma (mRCC), finding great tumour shrinkage and outcomes in ‘flare-responders’ whose CRP levels initially increased but then decreased within three months to levels below baseline (16). Several other studies have demonstrated a correlation between early CRP kinetics and improved response in metastatic urothelial carcinoma (mUC) and non-small cell lung cancer (NSCLC). In addition, preoperative CRP has been identified as a potential biomarker for HCC patients who have received surgery, TKIs, and ICIs (17). However, the impact of early CRP kinetics on TACE combined with LEN and ICIs remains to be fully demonstrated in HCC patients.

In this study, we explored the association between early CRP kinetics and prognosis in HCC patients treated with TACE combined with LEN and ICIs (TACE–LEN–ICIs), in order to offer valuable insights for oncologists in their clinical practice.

## 2 Methods

### 2.1 Patients

HCC patients treated with TACE–LEN–ICIs between December 2020 and February 2024 were reviewed, and 143 of them were recruited into this retrospective cohort study. All the recruited patients were diagnosed based on non-invasive criteria or biopsy (18). Inclusion criteria were: (1) patients aged between 18 and 75 years; (2) Barcelona clinic liver cancer (BCLC) stage B or C; (3) Child–Pugh grade A or B and Eastern Cooperative Oncology Group (ECOG) 0 or 1; (4) initiation of LEN and ICIs within one month before or after TACE; and (5) at least one measurable target lesion according to the modified Response Evaluation Criteria in Solid Tumours (mRECIST) criteria.

Exclusion criteria were: (1) any history of treatments; (2) other malignancies; (3) immunodeficiency conditions; (4) severe dysfunction in heart, lung, liver, or kidneys; and (5) incomplete medical records.

The serum CRP level was measured prior to TACE–LEN–ICIs treatment, 1 month after treatment, and at least every 3 months thereafter. Then, patients were stratified into three subgroups: (i) CRP flare-responders, whose level increased to more than double the baseline value in 1 month after initiation of the TACE–LEN–ICIs regimen, then decreased to below baseline after 3 months; (ii) CRP responders, whose CRP levels decreased by at least 30% from baseline in 3 months with no initial ‘flare’; and (iii) CRP non-responders, who did not meet the criteria for the other two groups.

**Figure 1:**
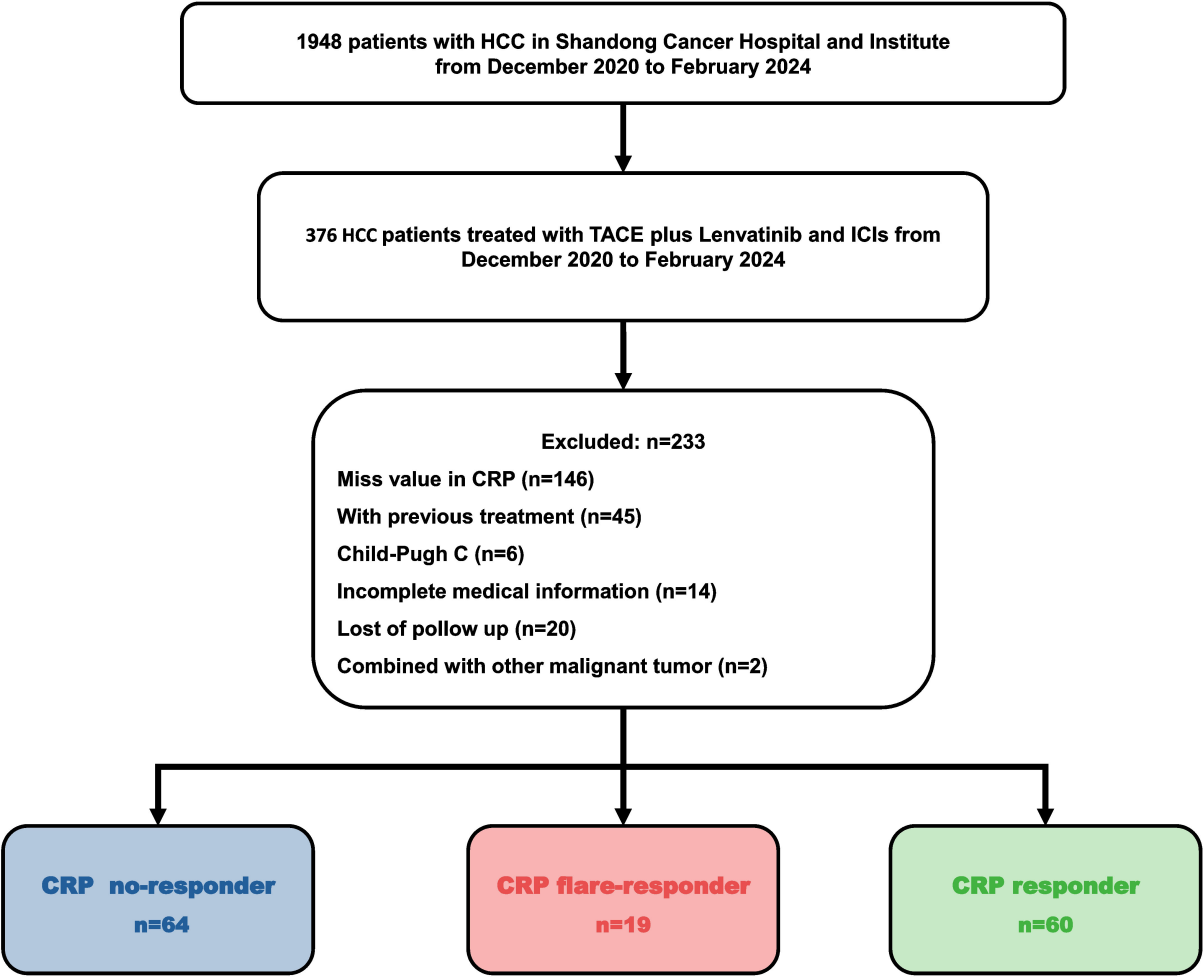
Flowchart of the patient selection process.

The Ethics Committee of Shandong First Medical University Affiliated Cancer Hospital approved this single-centre retrospective study (SDTHEC 2024004032). All patients gave written informed consent before initiating treatment. All procedures followed the 1955 Declaration of Helsinki. The recruitment process is shown in **Figure 1**.

### 2.2 TACE procedure

TACE was performed according to a previously reported protocol (19). Using the Seldinger technique, a 5 French catheter was inserted into the coeliac trunk, then a 2.7 French microcatheter was placed super-selectively in the blood supply artery. Microcatheter positioning was confirmed by angiography. The target artery was injected with 30 mg/m^2^ of epirubicin, lobaplatin 50 mg or raltitrexed added to 2–5 mL of lipiodol. Particulate embolic agents and up to 20 mL of lipiodol were injected to ensure the blood flow stagnated. This was considered the endpoint of embolisation. If there was a significant arteriovenous fistula, embolisation was performed. To embolise fistulas, large-diameter gelatin particles or spring coils are also used in addition to iodized oil. In order to ensure that all liver tumour lesions had been embolised, preoperative CT images were compared with those taken during embolisation. If embolisation was incomplete, extrahepatic blood supply vessels were detected and embolised. After two cycles of TACE, the imaging evaluation will determine whether TACE should be repeated. Subsequent ‘On-demand’ TACE were depended on the postoperative contrast-enhanced CT or MRI scans, along with tumour marker reassessment, to guide additional procedures.

### 2.3 Administration of LEN and ICIs

Patients were administered LEN 8 mg/day (if body weight <60 kg) or 12 mg/day (if body weight >60 kg), 3–7 days before TACE. The dose of LEN was adjusted (to 8 mg/day, or 4 mg/day, or 4 mg every other day), as necessary, in the event of LEN-related adverse events (AEs). An anti-PD-1 ICI (sintilimab, camrelizumab, or tislelizumab) was administered intravenously every three weeks, 0–1 day after the TACE procedure. Dose modification for these agents was also allowed due to AEs.

### 2.4 Follow-Up

Contrast-enhanced CT or dynamic MRI was conducted 4–6 weeks after the first TACE procedure, then every 3 months thereafter. Laboratory tests were conducted every 3 weeks. Two radiologists with 5 years of experience independently assessed the tumour response according to mRECIST guidelines. Patients were consistently follow-up until mortality or the conclusion of the study.

### 2.5 Outcomes

The primary outcomes were OS, defined as the period from initiation of therapy to death or the last visit, and PFS, the period from the first therapy to progression or death. Disease control rate (DCR) and ORR were also calculated.

### 2.6 Statistical analysis

Patient characteristics were summarised by medians and interquartile ranges (IQRs) for continuous variables with non-normal distribution, while categorical variables were described using frequencies and proportions. The χ^2^ test or Fisher’s exact test was used as appropriate. For continuous variables, one-way ANOVA was used for comparisons among three groups, while for comparisons between two groups, the t-test or Mann–Whitney U-test was applied as appropriate. Time-dependent receiver operating characteristic (ROC) curves were constructed to evaluate predictive accuracy. Cox’s proportional hazard regression analysis was performed for univariate and multivariate analysis of PFS and OS.

## 3 Results

### 3.1 Baseline characteristics

A total of 143 patients diagnosed with HCC between December 2020 and February 2024 and who met the inclusion and exclusion criteria were enrolled in this retrospective cohort study (**Figure 1**). The cohort included 121 (84.6%) males and 22 (15.4%) females. Consistent with the characteristics of HCC in China, the aetiology in the majority (91.6%) of cases was hepatitis B virus (HBV). Most patients were in BCLC stage C (86.7%) and Child–Pugh grade A (86.0%). About half of patients (53.7%) had macrovascular invasion, while 46.9% of cases presented with extrahepatic metastasis. Seventy-nine patients (55.2%) had an alpha-foetoprotein (AFP) level of over 400 ng/mL, and most subjects (74.1%) had a CRP level of 5 mg/L or more at baseline (**Table 1**). There were no significant differences between the groups in age, gender, aetiology, BCLC stage, and Child-Pugh grade (**Table 1**).

**Table 1:** Comparison of baseline parameters between CRP flare responders, CRP responders and CRP non-responders.

Characteristics		Overall	CRP flare-responders (N = 19)	CRP responders (N = 60)	CRP non-responders (N = 64)	*P-*value
Age (years)	<60	96 (67.1%)	12 (63.2%)	38 (63.3%)	46 (71.8%)	0.437
	≥60	47 (32.9%)	7 (36.8%)	22 (36.8%)	18 (28.2%)	
Gender	Female	22 (15.4%)	5 (26.4%)	8 (13.4%)	9 (14.1%)	0.369
	Male	121 (84.6%)	14 (73.6%)	52 (86.6%)	55 (85.9%)	
Child-Pugh	A	123 (86.0%)	18 (94.7%)	50 (83.3%)	55 (85.9%)	0.173
	B	20 (14.0%)	1 (5.3%)	10 (16.7%)	9 (14.1%)	
Etiology	Hepatitis B virus	131 (91.6%)	17 (89.4%)	56 (93.3%)	58 (90.6%)	0.048
	Others	12 (8.4%)	2 (10.5%)	4 (6.4%)	6 (9.4%)	
BCLC Stage	B	19 (13.3%)	3 (16.7%)	5 (8.3%)	11 (17.2%)	0.333
	C	124 (86.7%)	16 (84.2%)	55 (91.6%)	53 (82.8%)	
Max Size (cm)	<7.0	40 (28.0%)	5 (26.4%)	15 (25.0%)	20 (31.3%)	0.734
	≥7.0	103 (72.0%)	14 (73.6%)	45 (75.0%)	44 (48.7%)	
Number	Solitary	36 (25.2%)	6 (31.6%)	13 (21.7%)	17 (26.6%)	0.699
	Multiple	107 (74.8%)	13 (68.4%)	47 (78.3%)	47 (73.4%)	
Macrovascular Invasion	No	67 (46.9%)	9 (47.4%)	28 (46.7%)	30 (46.9%)	0.060
	Yes	76 (53.7%)	10 (52.6%)	32 (53.3%)	34 (53.1%)	
Extrahepatic Metastases	No	76 (53.7%)	9 (47.4%)	26 (46.7%)	41 (64.0%)	0.60
	Yes	67 (46.9%)	10 (57.6%)	34 (53.3%)	23 (36.0%)	
Line of therapy	1	88 (61.5%)	12(63.1%)	39 (65.0%)	37 (57.8%)	0.154
	2	55 (38.5%)	7 (36.9%)	21 (35.0%)	27 (42.2%)	
AFP (ng/ml)	< 400	64 (44.8%)	9 (47.4%)	27 (45.0%)	22 (34.4%)	0.068
	≥ 400	79 (55.2%)	10 (52.6%)	33 (55.0%)	42 (65.6%)	
CRP baseline (mg/L)	< 5	37 (25.8%)	9 (47.4%)	3 (5.0%)	25 (39.0%)	0.243
	≥ 5	106 (74.1%)	10 (52.6%)	57 (95.0%)	39 (61.0%)	
CRP baseline (mg/L)	Median, (IQR)	1.45(0.49-4.1)	0.51(0.32-1.25)	0.59(0.3-1.80)	3.77(2.10-6.21)	0.779
Albumin (g/dL)	Median, (IQR)	40.1(36.2-43.4)	42.4(40.1-44.2)	42(36.75-44.35)	38.1(35.1-41.58)	0.007
Total bilirubin (mg/dL)	Median, (IQR)	16.3(12.1-26.5)	13.1(11.5-20.3)	17.4(12.0-27.9)	16.5(12.4-28.68)	0.150
Platelet count(×10^9^/L)	Median, (IQR)	163(112-235)	176(130-217)	137(93-168.75)	208(160-266)	0.000
LDH	Median, (IQR)	262(218-350)	235(213-293)	247(216.25-304.5)	297(233.25-388.75)	0.015
PLR	Median, (IQR)	141(93.68-197.58)	136.21(82.48-181.58)	111.31(72.09-182.08)	159.05(119.22-200.85)	0.010
NLR	Median, (IQR)	3.07(2.18-4.53)	2.92(2.51-4.45)	2.84(1.76-3.98)	3.45(2.63-5.09)	0.038
PNI	Median, (IQR)	46(42.25-50.25)	48.2(43.65-51.9)	46.25(41.13-50.5)	45.43(42.18-47.19)	0.088
SII	Median, (IQR)	516.91(278.64-950.48)	578.52(304.59-759.6)	311.13(150.32-675.52)	764.54(425.71-120.898)	0.000

BCLC Stage, Barcelona clinic liver cancer stage; AFP, alpha-fetoprotein; CRP, C-reactive protein; LDH, lactatedehydrogenase; PLR, platelet to lymphocyte ratio; NLR, neutrophil to lymphocyte ratio; PNI, prognostic nutritional index; SII, systemic immune-inflammation index; IQR, interquartile range.

### 3.2 LEN and ICI treatment

All patients took LEN initially at 8 mg/day or 12 mg/day. Dose adjustments were made in 3 (15.8%) patients in the CRP flare-responder group, 12 patients (20%) in the CRP responder group, and 19 patients (26.7%) in the CRP non-responder group (*p* = 0.305) (**Supplementary Table S1**). Three ICI drugs were used in this study, namely sintilimab, camrelizumab, and tislelizumab (**Supplementary Tables S2**, **S3**). There were 11 patients treated with camrelizumab, 4 with sintilimab, and 4 with tislelizumab among the CRP flare-responder group. A total of 35 patients were treated with camrelizumab, 14 with tislelizumab, and 11 with sintilimab in the CRP responder group. Among the CRP non-responder group, 34 patients were treated with camrelizumab, 18 with tislelizumab, and 12 with sintilimab.

### 3.3 Early CRP kinetics

**Figure 2:**
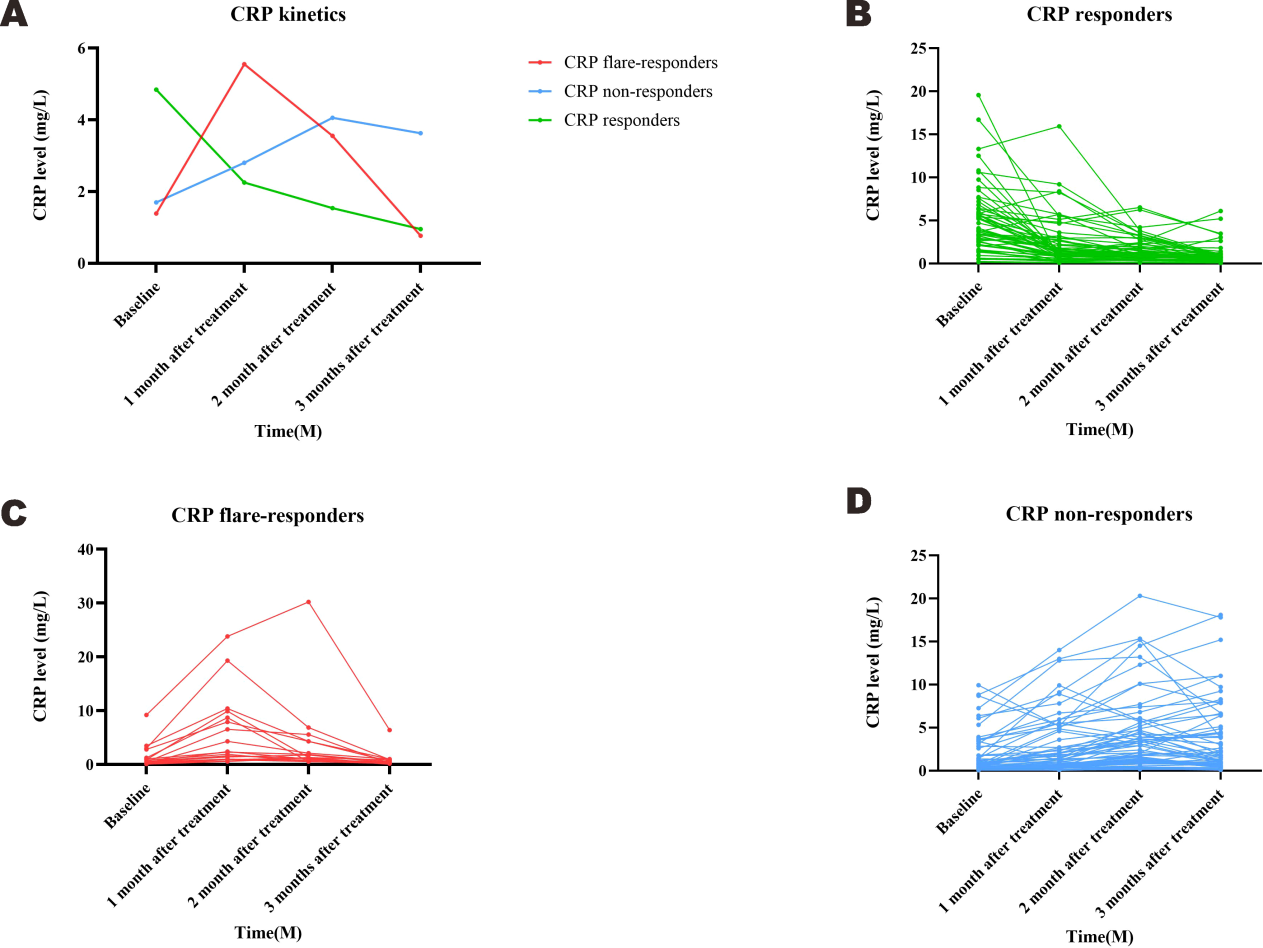
The variation curve of CRP kinetics in each group.

Based on measured trends in CRP levels, 19 patients were assigned into the CRP flare-responder group, 60 into the CRP responder group, and the remaining 64 into the CRP non-responder group. Patient characteristics for the three groups are summarised in **Table 1**, while details of the observed CRP kinetics are shown in **Figure 2**, which includes both group averages and individual profiles. The median CRP baseline level was 0.51 (IQR: 0.32–1.25) mg/L for the CRP flare-responder group, 0.59 (IQR: 0.30–1.81) mg/L for the CRP responder group, and 3.77 (IQR: 2.10–6.21) mg/L for the CRP non-responder group (**Table 1**).

### 3.4 Survival assessment

**Figure 3:**
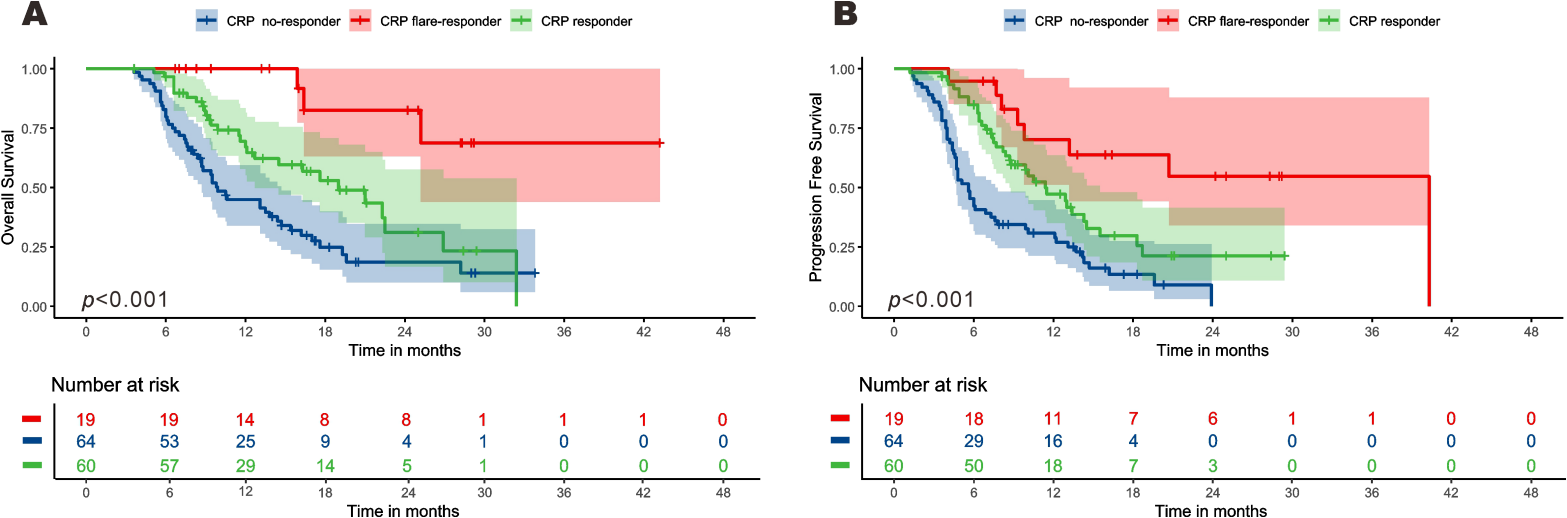
Kaplan–Meier curve for overall survival **(A)** and progression-free survival **(B)** among the three groups.

Survival times of the three CRP kinetics groups were compared. Among the CRP non-responder group, median PFS and OS were 5.5 [95% confidence interval (CI): 4.7–5.6] months and 9.9 (8.7–14.7) months respectively, compared with 11.4 (8.7–15.5) and 18.9 [12.7–not reached (NR)] months for the CRP responder group, and 40.3 (13.1–NR) and NE (25.2–NR) months for the CRP flare-responder group (**Figure 3**). Among the whole cohort, median PFS and OS were 8.1 (7.1-9.2) months and 16.2 (13.1-19.30 months, respectively.

The median follow-up time of the CRP non-responder group was 20.3 (15.5-25.0) months, that of the CRP responder group was 16.6 (15.8-17.4) months, and that of the CRP flare-responder group was 24.2 (13.0-35.5) months. Among the entire cohort, the median follow-up time was 18.2 (15.2-21.1) months.

### 3.5 Tumour response

Tumour response rates are shown in **Table 2**. The ORR for the CRP non-responder group, CRP responder group, and CRP flare-responder group was 48.4%, 78.4% and 78.9%, respectively (*p* = 0.001). In terms of DCR, this was observed as 78.1%, 96.7% and 94.8%, respectively, among the CRP non-responder group, CRP responder group and CRP flare-responder group (*p* = 0.004).

**Table 2:** Tumour response according to the CRP kinetics.

Variable	CRP flare-responders (N = 19)	CRP responders (N = 60)	CRP non-responders (N = 64)	*P*-value
CR	3 (15.8%)	4 (6.7%)	5 (7.8%)	
PR	12 (63.2%)	43 (71.7%)	26 (40.6%)	
SD	3 (15.8%)	11 (18.3%)	19 (29.7%)	
PD	1 (5.2%)	2 (3.3%)	15 (23.4%)	
ORR	15 (78.9%)	47 (78.4%)	31 (48.4%)	0.001
DCR	18 (94.8%)	58 (96.7%)	50 (78.1%)	0.004

CR, complete response; PR, Partial response, SD, stable disease; PD, progressive disease; ORR, objective response rate; DCR, disease control rate.

### 3.6 Prognostic factors for OS and PFS

We conducted univariate and multivariate Cox proportional hazards regression analysis to assess early CRP kinetics as a prognostic indicator for OS and PFS, as displayed in **Table 3**. In univariate analysis, PFS was significantly associated with AFP level [Hazard ratio (HR) = 1.731(1.148–2.612), *p* = 0.009], multiple tumours [HR = 1.762(1.078–2.878), *p* = 0.024], Child–Pugh grade B [HR = 2.066(1.197–3.566), *p* = 0.009], CRP responder status[HR = 0.479(0.313–0.732), *p* = 0.001] and CRP flare-responder status [HR = 0.194(0.087–0.432), *p* < 0.001]. Meanwhile, multivariate analysis identified multiple tumours [HR = 1.894(1.150–3.120), *p* = 0.012], Child–Pugh grade B [HR = 1.800(1.006–3.221), *p* = 0.048], CRP responder status [HR = 0.315(0.192–0.518), *p* < 0.001] and CRP flare-responder status [HR = 0.186(0.082–0.419), *p* < 0.001] as independent risk factors for PFS.

**Table 3:** Univariate and multivariate analyses factors associated with progression free survival and overall survival.

Variables		Progression-free survival				Overall survival			
		Univariate Analysis		Multivariate Analysis		Univariate Analysis		Multivariate Analysis	
		HR (95% CI)	*P-*value	HR (95% CI)	*P-*value	HR (95% CI)	*P-*value	HR (95% CI)	*P-*value
Age (years)	<60 *vs.* ≥60	1.164 (0.762-1.778)	0.482			1.164 (0.727-1.863)	0.527		
Immunotherapy	First line *vs.* Second line	1.119 (0.749-1.671)	0.583			1.029 (0.658-1.608)	0.901		
Gender	Male *vs.* Female	1.209 (0.707-2.069)	0.488			1.014 (0.558-1.843)	0.964		
BCLC Stage	B	Reference				Reference			
	C	1.273 (0.679-2.386)	0.452			1.223 (0.628-2.380)	0.554		
AFP	<400 *vs.* ≥400	1.731 (1.148-2.612)	0.009	0.965 (0.571-1.631)	0.894	1.533 (0.970-2.420)	0.067	0.483 (0.258-0.905)	0.023
Number Max Size (cm)	Solitary *vs.* Multiple	1.762 (1.078-2.878)	0.024	1.894 (1.150-3.120)	0.012	1.538 (0.887-2.668)	0.125	1.659 (0.930-2.959)	0.086
	<7 *vs.* ≥7	1.144 (0.730-1.794)	0.557			1.607 (0.947-2.726)	0.079	1.563 (0.875-2.791)	0.131
Extrahepatic Metastases	No *vs.* Yes	1.231 (0.825-1.836)	0.309			1.102(0.704-1.726)	0.617		
Macrovascular Invasion	No *vs.* Yes	1.039 (0.697-1.549)	0.851			1.341 (0.854-2.104)	0.202		
Child-Pugh	A	Reference		Reference		Reference			
	B	2.066 (1.197-3.566)	0.009	1.800 (1.006-3.221)	0.048	2.306 (1.302-4.081)	0.004	1.899 (1.028-3.509)	0.041
Hepatitis B virus	No *vs.* Yes	1.056 (0.548-2.036)	0.870			1.179 (0.541-2.567)	0.679		
CRP kinetics	CRP no-responder	Reference		Reference		Reference			
	CRP responder	0.479 (0.313-0.732)	0.001	0.315 (0.192-0.518)	<0.001	0.507 (0.317-0.810)	0.005	0.249 (0.140-0.441)	<0.001
	CRP flare-responder	0.194 (0.087-0.432)	<0.001	0.186 (0.082-0.419)	<0.001	0.113 (0.035-0.365)	<0.001	0.094 (0.028-0.313)	<0.001

BCLC Stage, Barcelona clinic liver cancer stage; AFP, alpha-fetoprotein; CRP, C-reactive protein; HR, hazard ratio; CI, confidence interval.

Univariate analysis showed that Child–Pugh grade B [HR = 2.306(1.302–4.081), *p* = 0.004], CRP responder status [HR = 0.507(0.317–0.810), *p* = 0.005], and CRP flare-responder status [HR = 0.113(0.035–0.365), *p* < 0.001] were significantly associated with OS. Multivariate analysis identified AFP level [HR = 0.483(0.258–0.905), *p* = 0.023], Child–Pugh grade B [HR = 1.899(1.023–3.509), *p* = 0.041], CRP responder status [HR = 0.249(0.140–0.441), *p* < 0.001], and CRP flare-responder status [HR = 0.094(0.028–0.313), *p* < 0.001] as independent risk factors for OS.

### 3.7 The effect of CRP kinetics on PFS and OS by subgroup

**Figure 4:**
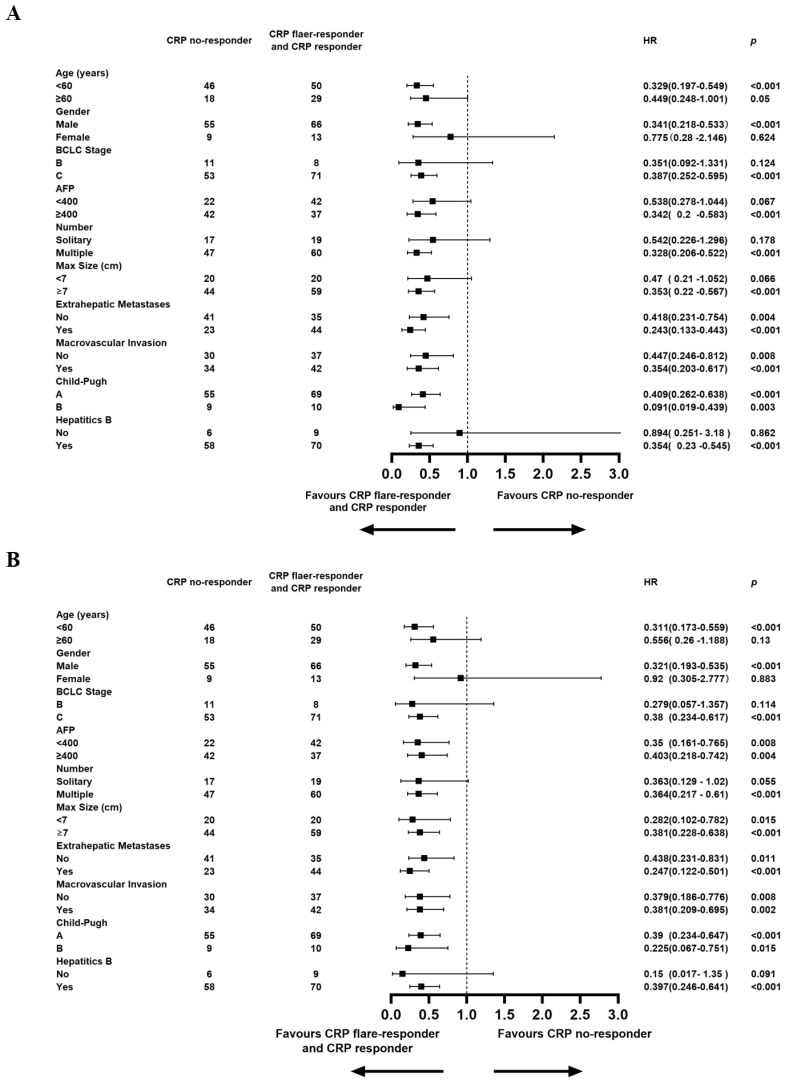
Median progression-free survival **(A)**, Median overall survival **(B)**, and hazard ratios (HR) for death comparing CRP Kinetics (CRP no-responder vs. CRP flaer-responder and CRP responder) in different subgroups in the entire cohort.

A detailed subgroup analysis was conducted, stratifying each variable to investigate the effect of CRP kinetics on the outcomes of TACE–LEN–ICIs. The results, depicted in **Figure 4A**, indicated that the CRP flare-responder and CRP responder group consistently exhibited superior PFS across various subgroups. Additionally, **Figure 4B** shows that the CRP flare-responder and CRP responder group displayed improved OS compared to the CRP no-responder group within various subgroups. In these subgroups, patients varied in age, gender, BCLC B or C stage, AFP level, single or multiple lesions, maximum tumour size, metastases, macrovascular invasion, Child-Pugh A or B, and hepatitis B virus (HBV).

### 3.8 Comparison of CRP kinetics with other prognostic scores

**Figure 5:**
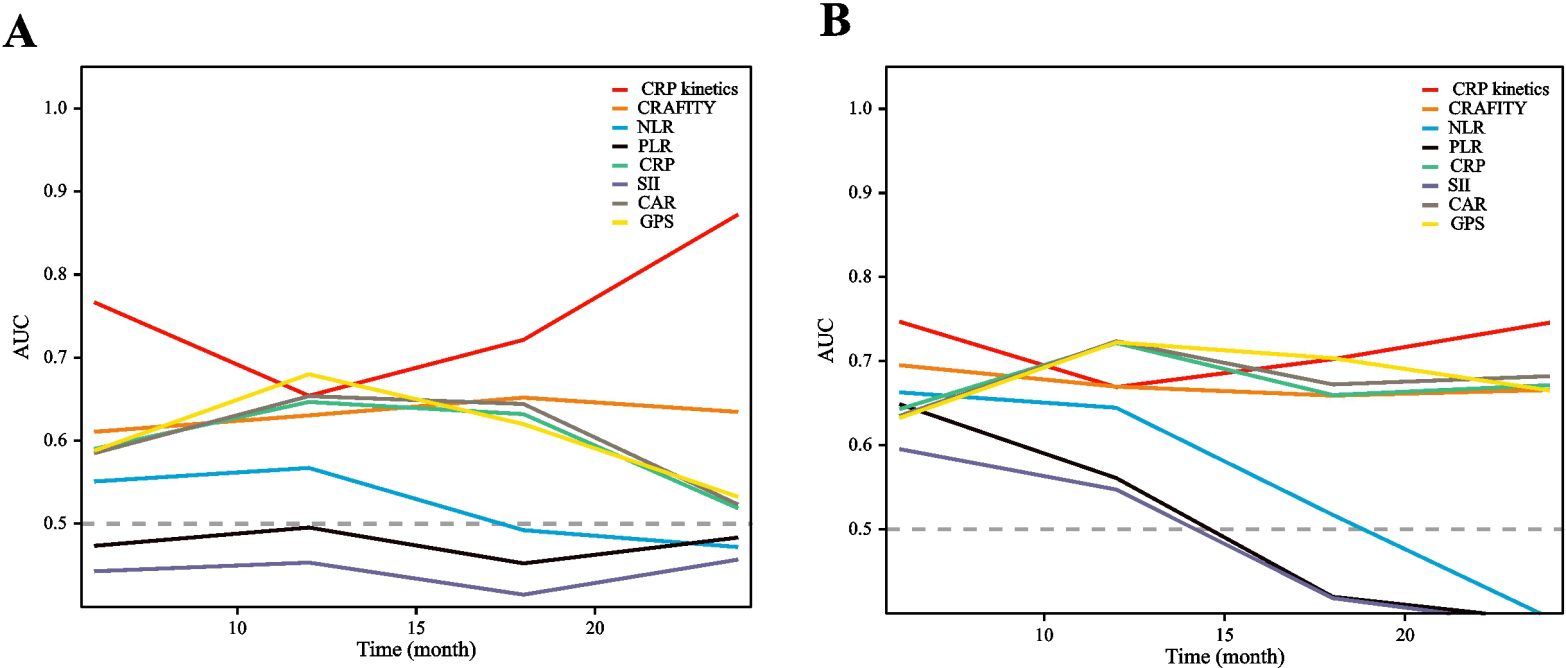
Time-dependent ROC curves of progression-free survival **(A)** and overall survival **(B)**.

Time-dependent receiver operating characteristic (ROC) curves were constructed to evaluate the predictive value of CRP kinetics compared with other inflammation markers, including the C-reactive protein and alpha fetoprotein in immunotherapy (CRAFITY) score, neutrophil–lymphocyte ratio (NLR), platelet-to-lymphocyte ratio (PLR), systemic immune inflammation index (SII), CRP-to-albumin ratio (CAR), prognostic nutritional index (PNI), and glasgow prognostic score (GPS) (detailed in **Supplementary Materials**). Based on these results, CRP kinetics model could more accurately predict OS and PFS of HCC patients treated with TACE–LEN–ICIs than the other markers considered (**Figure 5**). The area under the ROC curve (AUROC) values of the CRP kinetics model for PFS prediction at 12, 18, and 24 months were 0.654, 0.721, and 0.872, while for OS prediction were 0.669, 0.702, and 0.745, respectively (**Supplementary Table S4**).

### 3.9 Adverse events

Details of treatment-related AEs are listed in **Supplementary Table S1**. All AEs were manageable. No toxicities related deaths were observed during follow-up. Hypertension, vomiting, hypothyroidism, and liver injury were the most common AEs.

## 4 Discussion

In this retrospective study, we validated that early CRP kinetics is a promising predictive biomarker for HCC treated with the TACE–LEN–ICIs combination regimen. To our knowledge, this is the first report assessing the prognostic utility of a CRP kinetics model in HCC patients. This is significant because the majority of HCC cases in China are diagnosed at advanced stages, where high tumour burden and portal vein tumour thrombus (PVTT) often manifest as prevalent features due to the insidious onset of the disease. Unfortunately, these cases have a remarkably unfavourable prognosis, meaning that Chinese doctors are more inclined to use more aggressive treatment approaches with higher intensity such as the TACE–LEN–ICIs regimen investigated here. In fact, the combination of TACE with ICIs plus TKIs is a commonly used treatment strategy for patients with advanced HCC in China. As indicated by the CHANCE001 study, although TACE combined with PD-(L)1 inhibitors and MTT showed better PFS and OS than TACE monotherapy, around 40% of patients still fail to achieve an objective response (10). So, there is still an urgent need to find a concise and effective biomarker to predict the effectiveness of triple therapy.

The acute-phase protein CRP is well-recognised as an indicator of cancer-induced systemic inflammation, which is often evident in clinical symptoms (20). A multi-institutional study revealed a significant correlation between baseline CRP levels and prognosis in patients with HCC undergoing LEN treatment. Specifically, a baseline CRP level above 0.5 mg/dL was determined to be an unfavourable prognostic factor in HCC patients treated with LEN (21). Another study found that elevated CRP levels may indicate aggressive cancer progression and potential resistance to therapy with the ICI atezolizumab in combination with bevacizumab (17). Hence, it has been demonstrated that an elevated baseline concentration of CRP before oncological treatment is associated with worse clinical outcomes in HCC patients. Unfortunately, however, the dynamics of early changes in systemic inflammation after therapeutic intervention have mostly been neglected.

Recently, Fukuda et al. (16) described the CRP ‘flare-response’ phenomenon, characterised by an early increase in CRP after initial treatment of ICIs, followed by a subsequent reduction below baseline. It is noteworthy that this novel concept can correctly predict therapeutic success in 42 mRCC patients who received a PD-1 inhibitor. To date, here is substantial evidence to support the ability of CRP kinetics to rationalise the therapeutic effect of ICIs has been widely validated in mRCC (22), NSCLC (23), and mUC (24). However, there has been demonstrated a correlation between CRP kinetics and therapeutic response in HCC patients, so this retrospective study aimed to address the knowledge gap on CRP kinetics in the HCC field.

In total, 143 patients treated with TACE–LEN–ICIs were enrolled into the study. Based on early CRP kinetics, 19 patients were assigned as CRP flare-responders, 60 patients as CRP responders, and 64 patients as CRP non-responders. After initiating triple therapy, the median PFS of CRP flare-responders, CRP responders, and CRP non-responders was 40.3, 11.4, and 5.5 months, respectively (*p* < 0.001). The median OS of the same three groups was NE (not estimable), 18.9 months, and 9.9 months, respectively (*p* < 0.001). Thus, in this study, OS and PFS varied significantly across the three groups according to CRP kinetics. Because of its easy accessibility in clinical practice, CRP kinetics could be easily adopted in routine practice and serve as a valuable tool for oncologists in clinical decision-making.

Furthermore, recently published results from LEAP-012 demonstrated that for intermediate-stage HCC, TACE combined with lenvatinib and pembrolizumab significantly prolonged PFS (14·6 months) compared with TACE alone (25). Notably, 86.7% of patients in our study were classified as BCLC stage C. The median PFS and OS in the CRP flare-responders group were significantly superior to the results of LEAP-012. However, both the median PFS and OS in CRP responders and CRP non-responders were shorter than those reported in LEAP-012. These findings indicate that through effective biomarker screening, even patients with more advanced stages can achieve favourable treatment outcomes. This further underscores the predictive value of CRP kinetics, which can assist in our clinical decision-making.

Consistently, HCC patients characterised as CRP flare-responders or CRP responders experienced higher ORR (78.9% or 78.4% versus 48.4% for non-responders) and DCR (94.8% or 96.7% versus 78.1% for non-responders) than those with CRP non-responders in the whole cohort. The fact that only 48.4% of CRP non-responders responded to TACE–LEN–ICIs, which means CRP kinetics could identify such patients early. Alternative locoregional treatments such as salvage radiotherapy, hepatic arterial infusion chemotherapy (HAIC), drug-eluting beads transarterial chemoembolisation (D-TACE), or selective intra-arterial radiotherapy (SIRT) are also effective.

There has been a growing body of research on inflammation-based prognostic scores have been developed in recent years, including the NLR (26, 27), PLR (28), SII (29), CAR (30), prognostic nutritional index (PNI) (20), and CRAFITY score (31, 32). These scores were proven to perform acceptably in predicting cancer prognosis. To further assess the early CRP kinetics model, the present study performed direct comparisons of the prognostic scores above with CRP kinetics. The AUROC values of the CRP kinetics model for PFS prediction at 12, 18, and 24 months were 0.654, 0.721, and 0.872, while for OS prediction were 0.669, 0.702, and 0.745, respectively. Our results suggested that the CRP kinetics model may predict OS and PFS more accurately than other inflammation markers for HCC patients treated with the TACE–LEN–ICIs regimen. Although CRP kinetics has exhibited superiority in predicting therapeutic efficacy in the present study, the explanation for its correlation with the immunological underpinnings of HCC remains insufficient. To further facilitate the utility of CRP kinetics in patient stratification and the prediction of treatment responses in future clinical practice, additional studies are required to investigate the potential associations among baseline CRP levels, distinct post-immunotherapy CRP kinetic patterns, and the composition of the tumour microenvironment.

Since CRP is regularly used to monitor systemic infections and inflammatory status, it is essential to expand oncologists’ awareness of CRP kinetics in triple therapy. While acute local inflammation may occur in the liver after a TACE procedure, leading to an increase in white blood cells and CRP, a previous investigation found no remarkable destructive change along with minimal to absent inflammatory cell infiltration in the surrounding non-tumoural liver parenchyma at 14 days post-surgery (33). Therefore, we reasonably speculate that TACE will not affect the serum CRP level 1 month after the procedure. Our clinical experience also indicates that the inflammatory response in the body generally disappears by this time, so we consider it reasonable to believe that TACE has little effect on CRP level after 1 month. In addition, a rapid CRP increase during initiation of ICIs should not be misinterpreted as indicative of systemic infection. In our centre, we regularly monitor procalcitonin and clinical symptoms to determine whether infection is present. Nevertheless, further studies are necessary to certificate whether complementing CRP with other markers, including procalcitonin, might facilitate differentiation between immunotherapy responses, systemic infections, or TACE-induced systemic infections.

This study has four main limitations. First, selection bias cannot be avoided in a single-centre retrospective cohort study. Second, the total sample size was limited, especially in the CRP flare-responder group, which may restrict the statistical power of subgroup analyses. Third, the follow-up period was relatively short, which may compromise the reliability of survival analyses. Fourth, as the majority of our patients were positive for HBV, our model may be more applicable to Chinese populations. Thus, the model requires testing with large, multicentre cohorts based on different populations to further validate its performance.

## 5 Conclusion

Early CRP kinetics may have predictive value for prognosis in HCC patients undergoing TACE–LEN–ICIs regimen. A large scale prospective randomised controlled clinical trial is still needed to confirm this conclusion in the future.

## Data Availability

The raw data supporting the conclusions of this article will be made available by the authors, without undue reservation.
