# Supplementary material for: C-reactive protein flare: a promising prognostic predictor for patients with hepatocellular carcinoma treated with TACE combined with lenvatinib and immune checkpoint inhibitors
Source: Front Immunol. 2025 Nov 11;16:1657733. doi: 10.3389/fimmu.2025.1657733 (PMC12643971; doi:10.3389/fimmu.2025.1657733)
Supplement: Supplementary file 1 [file Table1.docx]

**SUPPLEMENTARY MATERIAL**

**Contents of Supplementary Appendix**

[**Tabel S1. Treatment-related adverse events according to the CRP kinetics model. 3**](#_Toc208942266)

[**Table S2. Immune checkpoint inhibitors agents. 4**](#_Toc208942267)

[**Table S3. Category and dosage of immune checkpoint inhibitors. 4**](#_Toc208942268)

[**Table S4. The AUCs of the CRP-kinetics model and other inflammation markers by time-dependent ROC analysis. 5**](#_Toc208942269)

[**Other inflammation markers 6**](#_Toc208942270)

[**CRAFITY score (1) 6**](#_Toc208942271)

[**NLR (neutrophil to lymphocyte ratio) (2) 6**](#_Toc208942272)

[**PLR (platelet to lymphocyte ratio) (2) 6**](#_Toc208942273)

[**CAR (C-reactive protein to albumin ratio) (3) 6**](#_Toc208942274)

[**GPS (Glasgow prognostic score) (4) 6**](#_Toc208942275)

[**SII (systemic Immune-inflammation Index) (5) 6**](#_Toc208942276)

[**References 7**](#_Toc208942277)

## Tabel S1. Treatment-related adverse events according to the CRP kinetics model.

| **Items** | **CRP flare-responders**  **(N=19)** | **CRP responders**  **(N=60)** | **CRP non-responders**  **(N=64)** | ***P*-value** |
| --- | --- | --- | --- | --- |
| Hypertension |  |  |  |  |
| Any | 8(42.1%) | 28(46.7%) | 26(40.6%) | 0.789 |
| Grade≥3 | 2(10.5%) | 5(8.3%0 | 6(9.4%) | 0.953 |
| Rash |  |  |  |  |
| Any | 2(10.5%) | 10(16. 7%) | 13(20.3%) | 0.6 |
| Grade≥3 | 0 | 2(3.3%) | 2(3.1%) | 0.728 |
| Vomiting |  |  |  |  |
| Any | 7(36.8%) | 23(38.3%) | 29(45.3%) | 0.671 |
| Grade≥3 | 0 | 4(6. 7%) | 6(9.4%) | 0.368 |
| Fatigue |  |  |  |  |
| Any | 3(15.8%) | 24(40%) | 21(32.8%) | 0.148 |
| Grade≥3 | 0 | 3(5%) | 4(6.3%) | 0.54 |
| Weight loss |  |  |  |  |
| Any | 3(15.8%) | 15(25%) | 20(31.3%) | 0.382 |
| Grade≥3 | 1(5.3%) | 3(5%) | 6(9.4%) | 0.603 |
| Decreased appetite |  |  |  |  |
| Any | 5(26.3%) | 18(30%) | 28(43.8%) | 0.184 |
| Grade≥3 | 0 | 2(3.3%) | 8(12.5%) | 0.059 |
| Hypothyroidism |  |  |  |  |
| Any | 6(31.6%) | 12(20%) | 13(20.3%) | 0.381 |
| Grade≥3 | 2(10.5%) | 2(3.3%) | 2(3.1%) | 0.335 |
| Liver injury |  |  |  |  |
| Any | 6(31.58%) | 21(35%) | 32(32%) | 0.156 |
| Grade≥3 | 1(5.26%) | 6(10%) | 12(18.75%) | 0.194 |
| Immune-related hepatitis |  |  |  |  |
| Any | 2(10.53%) | 3(5%) | 5(7.81%) | 0.671 |
| Grade≥3 | 0 | 1(1.67%) | 1(1.56%) | 0.855 |
| Immune-related pneumonitis |  |  |  |  |
| Any | 2(10.53%) | 2(3.33%) | 3(4.69%) | 0.446 |
| Grade≥3 | 1(5.26%) | 0 | 1(1.56%) | 0.232 |
| Immune-related myocarditis |  |  |  |  |
| Any | 1(5.26%) | 3(5%) | 3(4.69%0 | 0.994 |
| Grade≥3 | 0 | 0 | 1(1.56%) | 0.537 |
| Dose adjustment of lenvatinib | 3(15.79%) | 12(20%) | 19(26.69%) | 0.305 |

## Table S2. Immune checkpoint inhibitors agents.

| **Immune checkpoint inhibitor** | **CRP kinetics** | | |
| --- | --- | --- | --- |
|  | **CRP flare-responders**  **(N=19)** | **CRP responders**  **(N=60)** | **CRP non-responders**  **(N=64)** |
| Camrelizumab | 11 | 35 | 34 |
| Tislelizumab | 4 | 14 | 18 |
| Sintilimab | 4 | 11 | 12 |

## Table S3. Category and dosage of immune checkpoint inhibitors.

| **Category** | **Dose (mg)** |
| --- | --- |
| Tislelizumab (Shanghai,baijishenzhou, iotechnology Co. LTD) | 200 |
| Sintilimab (Suzhou, xinda pharmaceutical Co.Ltd) | 200 |
| Camrelizumab (Suzhou,shengdiya Biological Medicine Co., LTD) | 200 |

## Table S4. The AUCs of the CRP-kinetics model and other inflammation markers by time-dependent ROC analysis.

| **Model** | **Progression-free survival** | | | **Overall survival** | | |
| --- | --- | --- | --- | --- | --- | --- |
|  | 12-Month AUROC | 18-Month AUROC | 24-Month AUROC | 12-Month AUROC | 18-Month AUROC | 24-Month AUROC |
| CRP-kinetics | 0.654 | 0.721 | 0.872 | 0.669 | 0.702 | 0.745 |
| CRAFITY | 0.630 | 0.651 | 0.634 | 0.669 | 0.659 | 0.665 |
| NLR | 0.567 | 0.492 | 0.471 | 0.644 | 0.517 | 0.398 |
| PLR | 0.495 | 0.452 | 0.483 | 0.561 | 0.419 | 0.391 |
| CRP | 0.646 | 0.631 | 0.518 | 0.721 | 0.659 | 0.671 |
| CAR | 0.653 | 0.414 | 0.523 | 0.723 | 0.672 | 0.682 |
| GPS | 0.679 | 0.679 | 0.532 | 0.722 | 0.703 | 0.665 |
| SII | 0.453 | 0.414 | 0.456 | 0.547 | 0.418 | 0.384 |

AUC, area under curve; ROC, receiver operating characteristic curve; AUROC, area under the receiver operating characteristic curve; CRAFITY: C‑reactive protein and alpha‑fetoprotein in immunotherapy; NLR, neutrophil to lymphocyte ratio; PLR, platelet to lymphocyte ratio; CRP, C‑reactive protein; CAR, C-reactive protein to albumin ratio; GPS, glasgow prognostic score; SII, systemic immune-inflammation index.

## Other inflammation markers

### CRAFITY score (1)

CRAFITY score was based on the alpha-fetoprotein (AFP) and C‑reactive protein (CRP) levels. In this score, one point was awarded for AFP >100 ng/mL and one for CRP >1 mg/mL, thus categorising the cohort into three groups scoring 0 (AFP <100 ng/mL and CRP <1 mg/mL), 1 (AFP >100 ng/mL or CRP >1 mg/mL) or 2 (AFP >100 ng/mL and CRP >1 mg/mL).

### NLR (neutrophil to lymphocyte ratio) (2)

NLR was calculated as the ratio of the absolute count of neutrophils (number of neutrophils/µL) to the absolute count of lymphocytes (number of lymphocytes/µL).

### PLR (platelet to lymphocyte ratio) (2)

PLR was calculated as the ratio of the absolute count of platelets (number of platelets/µL) to the absolute count of lymphocytes (number of lymphocytes/µL).

### CAR (C-reactive protein to albumin ratio) (3)

CAR was calculated by dividing the CRP level by the albumin level.

### GPS (Glasgow prognostic score) (4)

In GPS, patients with both an elevated CRP level (>1.0 mg/dl) and hypoalbuminemia (<3.5 g/dl) were allocated a score of 2, patients with only one of these biochemical abnormalities were allocated a score of 1, and patients with neither of these abnormalities were allocated a score of 0

### SII (systemic Immune-inflammation Index) (5)

SII = platelet count (×10^9/L) × neutrophil count (×10^9/L)/lymphocyte count (×10^9/L)

## References

1. Weijie W, Zhenyun Y, Hao Z, Teng L, Zhongguo Z, Yaojun Z, et al. The CRAFITY score emerges as a paramount prognostic indicator in hepatocellular carcinoma patients received Lenvatinib and Pembrolizumab. *Front Immunol* 2024;**15**(0) doi 10.3389/fimmu.2024.1474456.

2. Roberto M, Massimo V, Giuseppe G, Federico F, Andrea C, Filippo P*, et al.* A Multicenter International Retrospective Investigation Assessing the Prognostic Role of Inflammation-Based Scores (Neutrophil-to-Lymphocyte, Lymphocyte-to-Monocyte, and Platelet-to-Lymphocyte Ratios) in Patients with Intermediate-Stage Hepatocellular Carcinoma (HCC) Undergoing Chemoembolizations of the Liver. *Cancers (Basel)* 2024;**16**(9) doi 10.3390/cancers16091618.

3. Toshifumi T, Takashi K, Atsushi H, Masashi H, Kazuya K, Joji T*, et al.* C-reactive protein to albumin ratio predicts survival in patients with unresectable hepatocellular carcinoma treated with lenvatinib. *Sci Rep* 2022;**12**(1) doi 10.1038/s41598-022-12058-y.

4. Akiyoshi K, Hiroshi O, Nami I, Akira I, Mutumi O, Ken T*, et al.* The Glasgow Prognostic Score, an inflammation based prognostic score, predicts survival in patients with hepatocellular carcinoma. *BMC Cancer* 2013;**13**(0) doi 10.1186/1471-2407-13-52.

5. Shao-Ming W, Zhen-Xin Z, Rong-Jian P, Jia-Yi W, Jun-Yi W, Meng-Chao L*, et al.* Prognostic Value of the SII-PNI Score in Unresectable HCC Treated with Transcatheter Arterial Chemoembolization Combined with Lenvatinib and PD-1 Inhibitors. *J Inflamm Res* 2025;**18**(0) doi 10.2147/jir.S520339.
